# P01.02. Psychophysiological assessment of the impact of mind-body interventions—pilot trial to determine the best assessment methods

**DOI:** 10.1186/1472-6882-12-S1-P2

**Published:** 2012-06-12

**Authors:** R Glick, A White, C Greco, B Handen, E Szigethy, J Jennings

**Affiliations:** 1 University of Pittsburgh School of Medicine, Pittsburgh, USA

## Purpose

This study seeks an inexpensive, non-invasive, and easily reproducible measure of autonomic activity, which could greatly advance mind-body research and treatment. Specific aims of this study were to: explore the utility of selected physiological measures as a gauge of autonomic function; confirm the usefulness of Stroop and Math as stressors; and explore the utility of this testing protocol for changes over time.

## Methods

Subjects from three treatment studies were enrolled, assessing the impact of biofeedback, hypnosis, and MBSR. Pre and post treatment testing was conducted using Bionex hardware and software from MindWare Technologies. For one group, recordings were obtained with subjects watching a video-movie and for the other two during Stroop and Math Stressor tasks. Cardiovascular outcomes included Heart Rate Variability (HRV), impedance cardiography, and BP monitoring.

## Results

Three representative subjects, 1 from each group, were reviewed. The most consistent findings were an increase in the square root of the mean squared difference of successive RR-intervals (RMSSD) of 13.4%, 12.4%, and 41.7% for these 3 subjects; and decrease in BP by 11.2%, 6.4%, and 4.3% from pre to post recordings, reflecting an increase in parasympathetic and decrease in sympathetic activity following the intervention. High Frequency HRV (HF), Heart Rate, and Finger Pulse Amplitude (FPA) were also useful measures, while Pre-Ejection Period gave inconsistent values. We did not see a clear repeatable pattern of autonomic markers in response to the stressors.

## Conclusion

We found RMSSD and BP to be the most useful parasympathetic and sympathetic measures respectively. FPA may serve as an inexpensive continuous proxy for BP. Across these three interventions, we found a shift from pre to post treatment with increased parasympathetic and decreased sympathetic activity. Our stressors yielded inconsistent responses, but the average values over the 30 minute testing period were useful for assessing treatment effect. Psychophysiological testing is potentially of great utility in understanding the mechanism of mind-body interventions.

